# Early Onset of SARS-COV-2 Antibodies after First Dose of BNT162b2: Correlation with Age, Gender and BMI

**DOI:** 10.3390/vaccines9070685

**Published:** 2021-06-22

**Authors:** Raul Pellini, Aldo Venuti, Fulvia Pimpinelli, Elva Abril, Giovanni Blandino, Flaminia Campo, Laura Conti, Armando De Virgilio, Federico De Marco, Enea Gino Di Domenico, Ornella Di Bella, Simona Di Martino, Fabrizio Ensoli, Diana Giannarelli, Chiara Mandoj, Valentina Manciocco, Paolo Marchesi, Francesco Mazzola, Silvia Moretto, Gerardo Petruzzi, Fabrizio Petrone, Barbara Pichi, Martina Pontone, Jacopo Zocchi, Antonello Vidiri, Branka Vujovic, Giulia Piaggio, Aldo Morrone, Gennaro Ciliberto

**Affiliations:** 1 Department Otolaryngology Head and Neck Surgery, IRCCS Regina Elena National Cancer Institute, Istituti Fisioterapici Ospitalieri (IFO), 00144 Rome, Italy; raul.pellini@ifo.gov.it (R.P.); flaminiacampo@gmail.com (F.C.); valentina.manciocco@ifo.gov.it (V.M.); Paolo.marchesi@ifo.gov.it (P.M.); francesco.mazzola@ifo.gov.it (F.M.); silvia.moretto@ifo.gov.it (S.M.); gerardo.petruzzi@ifo.gov.it (G.P.); barbara.pichi@ifo.gov.it (B.P.); jacopo.zocchi@ifo.gov.it (J.Z.); 2 HPV Unit, UOSD Tumor Immunology and Immunotherapy, IRCCS Regina Elena National Cancer Institute, Istituti Fisioterapici Ospitalieri (IFO), 00144 Rome, Italy; 3 Department of Microbiology and Virology, IRCCS San Gallicano Dermatological Institute, Istituti Fisioterapici Ospitalieri (IFO), 00144 Rome, Italy; fulvia.pimpinelli@ifo.gov.it (F.P.); elva.abril@ifo.gov.it (E.A.); enea.didomenico@ifo.gov.it (E.G.D.D.); fabrizio.ensoli@ifo.gov.it (F.E.); martina.pontone@ifo.gov.it (M.P.); 4 Oncogenomic and Epigenetic Unit, IRCCS Regina Elena National Cancer Institute, Istituti Fisioterapici Ospitalieri (IFO), 00144 Rome, Italy; giovanni.blandino@ifo.gov.it; 5 Department Clinical Pathology and Cancer Biobank, IRCCS Regina Elena National Cancer Institute, Istituti Fisioterapici Ospitalieri (IFO), 00144 Rome, Italy; laura.conti@ifo.gov.it (L.C.); Chiara.mandoj@ifo.gov.it (C.M.); 6 Department of Biomedical Sciences, Humanitas University, Pieve Emanuele, 20089 Milan, Italy; armando.devirgilio@gmail.com; 7 Department of RiDAIT, IRCCS Regina Elena National Cancer Institute, Istituti Fisioterapici Ospitalieri (IFO), 00144 Rome, Italy; federico.demarco@ifo.gov.it; 8 Istituti Fisioterapici Ospitalieri (IFO), 00144 Rome, Italy; ornella.dibella@ifo.gov.it (O.D.B.); branka.vujovic@ifo.gov.it (B.V.); 9 Department of Pathology, IRCCS Regina Elena National Cancer Institute, Istituti Fisioterapici Ospitalieri (IFO), 00144 Rome, Italy; simona.dimartino@ifo.gov.it; 10 Biostatistical Unit, IRCCS Regina Elena National Cancer Institute, Istituti Fisioterapici Ospitalieri (IFO), 00144 Rome, Italy; diana.giannarelli@ifo.gov.it; 11 U.O.C. D.I.T.R.A.R. IRCCS Regina Elena National Cancer Institute, Istituti Fisioterapici Ospitalieri (IFO), 00144 Rome, Italy; fabrizio.petrone@ifo.gov.it; 12 Department of Radiology and Diagnostic Imaging, IRCCS Regina Elena National Cancer Institute, Istituti Fisioterapici Ospitalieri (IFO), 00144 Rome, Italy; antonello.vidiri@ifo.gov.it; 13 UOSD SAFU, IRCCS Regina Elena National Cancer Institute, Istituti Fisioterapici Ospitalieri (IFO), 00144 Rome, Italy; giulia.piaggio@ifo.gov.it; 14 Scientific Direction, IRCCS San Gallicano Dermatological Institute, Istituti Fisioterapici Ospitalieri (IFO), 00144 Rome, Italy; aldo.morrone@ifo.gov.it; 15 Scientific Direction, IRCCS Regina Elena National Cancer Institute, Istituti Fisioterapici Ospitalieri (IFO), 00144 Rome, Italy; gennaro.ciliberto@ifo.gov.it

**Keywords:** COVID-19, SARS-CoV-2, vaccine, obesity, antibodies, serum titer

## Abstract

Background: The first goal of the study was to analyse the antibody titre 21 days after the first dose of the BNT162b2 vaccine in a group of 252 healthcare workers (HCW). The second goal was to analyse how the antibody titre changes in correlation with age, gender and body mass index (BMI). Methods: Participants had a nasopharyngeal swab for SARS-CoV-2 and were assessed for the presence of SARS-CoV-2 antibodies at baseline and 21 days after the BNT162b2 priming dose. Results: First dose of BNT162b2 activated immune responses in 98% of the participants. Five HWC had no increase in antibody titre 21 days after the first dose. Antibody titre was greater in young (<38 years) vs. older participants (<38 vs. 47–56 *p* = 0.002; <38 vs. >56 *p* = 0.001). Higher antibody levels were detected in underweight vs. pre-obesity group (*p* = 0.026) and in normal-weight vs. pre-obesity group (*p* = 0.007). This association was confirmed after adjusting for age (*p* = 0.0001) and gender (*p* = 0.00001). Conclusions: Our study demonstrates that a single dose of BNT162b2 activates the immune response, and being young and normal-weight correlate positively with this response. Larger specifically designed clinical trials are needed to validate these results.

## 1. Introduction

Since the first cases of COVID-19 were described in December 2019, a health emergency with major social and economic disruptions has spread worldwide [1].

Control measures such as the use of masks, physical distancing and contact tracing helped to limit viral transmission; however, despite these valid measures, SARS-CoV-2 continues to spread [2].

Rapid production and deployment of vaccines represent the main viable road to limit the potential impact on populations and essential services [3].

A large clinical trial phase 2/3 with 44,000 people showed that a two-dose regimen of the vaccine BNT162b2, developed by BioNTech and Pfizer, has 95% efficacy in preventing symptomatic COVID-19. The same study showed that safety over a median of 2 months was similar to that of other viral vaccines [4].

Antibody titre could be used to predict protection against SARS-CoV-2, as already done for many viruses in humans and for SARS-CoV-2 in the animal challenge [5,6].

In this setting, it is interesting to know if a single dose can be effective in inducing antibody responses.

We report the early experience with BNT162b2 vaccination in healthcare workers (HCW). The first goal of our study was to analyse IgG antibody titre against Spike protein 21 days after the first dose of vaccine in a group of 252 HCW. Our second goal was to analyse if antibody responses differ in relation to age, gender and body mass index (BMI).

## 2. Materials and Methods

### 2.1. Study Design and Participants

A collaborative team carried out an immunogenicity evaluation among HCW vaccinated at the Istituti Fisioterapici Ospitalieri (IFO).

The study protocol complied with the tenets of the Helsinki declaration and was approved by the institutional scientific ethic committee (protocol RS1463/21), and the trial was registered with International Standard Randomised Controlled Trial Number (ISRCTN) 55371988.

All the enrolled participants met the following inclusion criteria: (1) provided written informed consent (2) age between 18–75 years, (3) health workers employed at the Istituti Fisioterapici Ospitalieri (IFO), (4) vaccinated at the Istituti Fisioterapici Ospitalieri (IFO). Key exclusion criteria included: (1) evidence of current or previous SARS-CoV-2 infection by either anamnesis, serological or microbiological test by nasopharyngeal swab before enrolment, (2) treatment with immunosuppressive therapy, (3) immunosuppression-associated pathology and (4) pregnancy.

Human SARS-CoV-2 infection convalescent sera (n = 59) were drawn from HCW donors (mean age 45) at least 14 days after PCR-confirmed diagnosis and at a time when the participants were asymptomatic. These sera were utilised as control for the test in previously infected HCW that is expected to have high levels of antibodies.

Manufacturer’s (BioNTech/Pfizer, Mainz, Germany) instructions for storage and administration of vaccine were followed. Briefly, COVID-19 mRNA Vaccine BNT162b2 was stored in an ultra-low temperature freezer at −80 °C. The undiluted vaccine was stored for up to 2 h at temperatures up to 25 °C, prior to use. The mRNA vaccine was administered as a 30 microgram/0.3 mL intramuscular injection into the deltoid muscle on day 1 of the study.

Participants had a nasopharyngeal swab for SARS-CoV-2 and were assessed for the presence of SARS-CoV-2 antibodies at baseline representing an unvaccinated population, and thereafter they received BNT162b2 vaccine. Twenty-one days after the BNT162b2 priming dose, nasopharyngeal swab and sera were collected. A questionnaire to collect data on the participants’ socio-demographic and health characteristics was administered. Participants were stratified by age, sex and body mass index (BMI).

**Assessment of SARS-CoV-2 in nasopharyngeal swab**

A nasopharyngeal swab was collected by standard procedures [7], and the presence of SARS-CoV-2 was determined by RT-PCR testing (Viracor, Eurofins Clinical Diagnostics, Lees Summit, MO, USA) following the manufacturer’s instruction.

**Assessment of SARS-CoV-2 Binding Antibodies**

Peripheral venous blood samples of 7–8 mL were obtained, serum collected and stored at +4 °C.

Measurement of IgG antibodies against S1/S2 antigens of SARS-CoV-2 was performed with a commercial chemiluminescent immunoassay (The LIAISON^®^ SARS-CoV-2 S1/S2 IgG test, Diasorin, Italy) according to manufacturer’s instruction. Positive and negative samples furnished by the manufacturer were run in parallel.

### 2.2. Statistical Analysis

Log Geometric Mean of AU/mL was reported. To assess differences between groups, Student’s *t*-test (Bonferroni’s adjusted) was used when comparing between 2 groups and ANOVA when comparing between >2 groups. Repeated measurement ANOVA was used to assess differences between groups over time.

Age was categorised according to quartiles. Statistical analysis was done using SPSS Statistics software version 21. A *p* < 0.05 was considered statistically significant.

## 3. Results

In total, 263 HCW gave written consensus to the study. According to the inclusion criteria, 11 HCW were excluded for previous SARS-CoV-2 infection. 

252 HCW were enrolled, 161 women (68.8%) and 91 men (36.2%). The mean age was 47 years (range 23–69).

Twenty-one days after the first dose, 98% of participants showed antigen-specific humoral response with respect to baseline levels, only five HCW had no response, and no one showed positive nasopharyngeal test.

**Figure 1:**
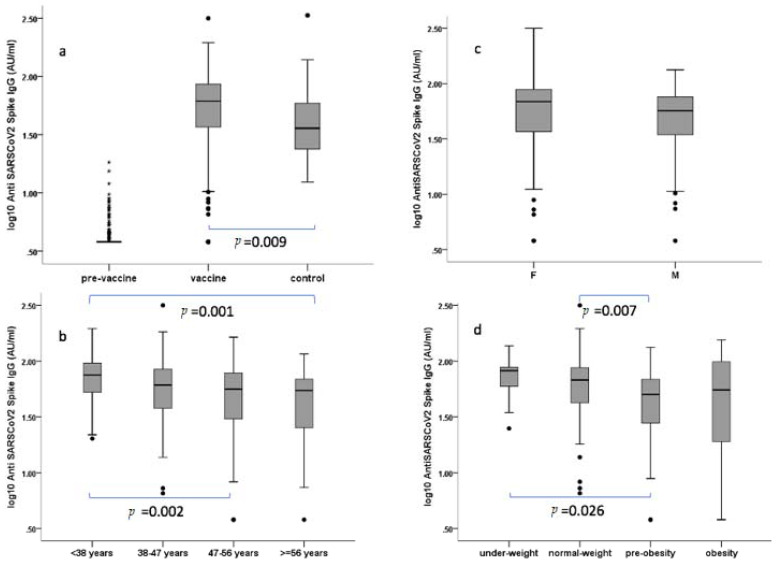
Levels of anti-SARS-CoV-2 spike IgG antibodies. Sera were analysed by LIAISON^®^ SARS-CoV-2 S1/S2 IgG test (Diasorin, Italy) as in methods. Positive (range 15–45 AU/mL) and negative controls (range <3.8–6.00 AU/mL) scored 31.6 AU/mL and <3.8 AU/mL, respectively. (**a**) pre-vaccine vs. vaccine vs. control group from previous infected HCW; (**b**) by gender (F: female, M: male); (**c**) by age classes; (**d**) by BMI classes. Serum was collected from the participant’s antibody 21 days after the priming dose. Antibody levels were expressed as log10 of concentration in Arbitrary Unit (AU). Age was categorised according to quartiles. Body mass index (BMI) classes were categorised according to Weir CB and Jan A [8]. Black dots represent outlier values.

Antibody concentrations ranged between 3.8–316 AU/mL that is similar to that of positive control sera from previous infected HCW (12.4–335 AU/mL). However, antibody geometric mean concentration (aGMC) (52.2 AU/mL, 95% CI: 47.6–57.2) was higher (*p* = 0.009) than that of these controls (39.4 AU/mL, 95% CI: 33.1–46.9) (Figure 1a). Paired tests between T0 and T1 were significant for all the variables (*p* < 0.0001). Results are summarised in Table 1.

Antibody titre was greater in young (<38 years) vs. older participants (<38 vs. 47–56 *p* = 0.002; <38 vs. >56 *p* = 0.001) (Figure 1b). Responses of greater magnitude were observed in women (55.8 AU/mL) vs. men (46.2 AU/mL) but was not statistically significant (*p* = 0.055) (Figure 1c), and a strong correlation (*p* = 0.001) was detected between aGMC and BMI, with higher antibody levels in the underweight vs. pre-obesity group (*p* = 0.026) and in the normal-weight vs. pre-obesity group (*p* = 0.007) (Figure 1d). This association was confirmed after adjusting for age (*p* = 0.0001) and gender (*p* = 0.00001).

A multivariate analysis accounting for potential confounding was performed by the inclusion of covariates. Data on multivariate linear regression of AU/mL are reported in Table 2. This analysis confirmed that age and BMI are statistically associated with differences in antibody response after vaccination, whereas gender has a lower significance level (*p* = 0.43).

## 4. Discussion

During the last year, we assisted in a remarkable effort by researchers and the pharmaceutical industry for the development of a vaccine against SARS-CoV-2. Although data on the safety and efficacy of the BNT162b2 vaccine demonstrate its effectiveness, immunogenicity data are reported only on small cohorts [9], and a phase 2/3 study on vaccine immunogenicity is ongoing [4]. Neutralizing antibodies are commonly accepted to be a functional biomarker of in vivo disease protection [10]. In our study, we used a chemiluminescent immunoassay that detected S1/S2 specific antibodies but was not specifically designed for neutralizing antibodies. However, the manufacturer indicates that with 80-AU/mL levels, the probabilities of having plaque reduction neutralization titres of 1:80 and 1:160 were 92% and 87%, respectively [11]. Thus, we can assume that at least a consistent part (30.9%) of the enrolled population showing >80 AU/mL should have developed neutralizing antibodies. This result is strengthened by a recent report on a cohort of 9109 vaccine-eligible HCWs. Indeed, authors showed substantial early reductions in SARS-CoV-2 infection and symptomatic COVID-19 rates following first vaccine dose administration [12].

Several recent reports indicate that a single dose of BNT162b2 is able to increase immune response even in people with pre-existing immunity [13,14,15,16]. In addition, another vaccine made with different technologies, the ChAdOx1 nCoV-19 (AZD1222) vaccine, showed the same immunogenicity and protection afforded by the first dose [17].

However, extended follow-ups to assess the long-term effectiveness of a single dose are needed to inform a second dose delay policy.

Although our study was on a limited number of subjects and cannot be assumed representative of the general population, collected data clearly shows that a single dose of BNT162b2 activates a humoral immune response. Only five participants did not show any increase in IgG levels. All these subjects were >47-year-old and had BMI > 25, ranging in the pre-obesity group. However, the low number cannot allow making a valid conclusion explaining this data.

Lean and younger people have statistically significant higher levels of antibody response compared to the overweight and older population. As the study was dealing with multiple variables, a multivariate analysis was done, and it confirmed the statistical significance for BMI and age (Table 2). It was suggested that obesity may hinder the COVID-19 vaccine and that immunosenescence developed while ageing may lead to a poor immune response to the vaccine [18].

Indeed, obesity can induce B cell defects with decreased frequency of regulatory B cell in association with higher frequencies of circulating Th1/Th17 cell [19]. In obese mice and humans, it has been demonstrated that adipose tissue (AT) B cells present antigen, secrete pro-inflammatory cytokines and chemokines, as well as autoimmune pathogenic antibodies [20]. In obese mice, activated splenocytes produce fewer anti-inflammatory cytokines and more B cell-derived pro-inflammatory cytokines in comparison with splenocytes from normal-weight mice [21]. This imbalance production of IL-6 and IL-10 was also confirmed in mitogen-stimulated B cells in individuals with obesity [22]. Other mechanisms impairing B cells functions are (i) the presence of pro-inflammatory microRNAs (miRs), in particular miR-155 and miRs-16 [23]; (ii) the secretion of Leptin by AT, which is able to induce secretion of pro-inflammatory cytokines by immune cells (i.e., macrophages, T or B cells) [22]; (iii) an increased level of saturated free fatty acid that is able to stimulate pro-inflammatory cytokine production in human [24]. The impairment of B cells function could induce a reduced response to vaccination. Obesity is also linked to less-diverse populations of microbes in the gut, nose and lungs, with altered compositions and metabolic functions compared with those in lean individuals. Recently, researchers reported that changes in the gut microbiome by taking antibiotics might alter responses to the flu vaccine [25]. Moreover, vaccines against influenza [22], hepatitis B [26], rabies [27] and tetanus [28] have shown reduced responses in those who are obese compared with those who are lean. A number of studies demonstrated BMI association with decreased serum response to vaccine during influenza vaccine season [29,30]. Immunosenescence associated with ageing is a known factor that may affect the immune response to a vaccine [18]. In older adults, a number of qualitative differences were observed in the B cell compartment, including class switch recombination, differentiation into plasma cells and expansion of a pro-inflammatory subset of B cell [31,32]. This subset of B cell is resembling [33,34]. In humans, it was shown that an increased level of CD27+ ABCs in the blood of the elderly was associated with a low titre of influenza antibodies [35].

In our cohort, the differences in IgG levels by age, gender and BMI are of unclear clinical significance in the absence of known correlates of protection and could be affected by the small size of samples. In addition, a further limitation is that no data of previous illnesses or medications were available. However, our study should be considered as an alarm bell for public and private research groups having larger datasets of anti-COVID19 vaccinated to carry out careful analyses on antibody responses. Finally, these associations have to be confirmed after a complete schedule of BNT162b2 vaccination.

## 5. Conclusions

Our data, together with that of Jabal et al. [13], seem to indicate that a single dose could produce an immune response even if there are no data on protection. For this reason, we cannot affirm that second dose delaying is a safe way in the race against faster-spreading variants of the virus, but it could be necessary. In particular, this strategy could be particularly useful in countries facing vaccine shortages and scarce resources, allowing higher population coverage with a single dose. However, only large specifically designed clinical trials could fully address this crucial question.

## Figures and Tables

**Table 1:** Confidence intervals by age, gender and BMI.

Characteristic	Number	GMC (95% CI)	GMC (95% CI)
SAMPLING		T0	T1
Age			
<38	63	4.08 (3.8–4.3)	70.23 (62.8–78.5)
≥38 < 47	63	4.26 (3.9–4.5)	56.33 (47.0–67.5)
≥47 < 56	67	4.13 (3.9–4.3)	44.05 (35.6–54.4)
≥56	59	4.03 (3.8–4.2)	42.37 (35.0–51.3)
Gender			
Female	161	4.02 (3.9–4.1)	55.82 (49.7–62.7)
Male	91	4.32 (4.0–4.6)	46.26 (39.8–53.8)
BMI			
Underweight	19	3.93 (3.7–4.1)	70.21 (57.2–86.1)
Normal Weight	148	4.14 (4.0–4.2)	58.44 (52.8–64.7)
Pre-obesity	59	4.10 (3.8–4.3)	40.39 (32.9–49.6)
Obesity	26	4.23 (3.7–4.7)	39.26 (25.5–60.4)

GMC: geometric mean concentration; CI: confidence interval; BMI: body mass index according to Weir CB and Jan A [8]. T0, samples from naïve population before the first vaccine dose; T1, samples 21 days after the first dose. Paired tests between T0 and T1 were significant for all the variables (*p* < 0.0001).

**Table 2:** Multivariate linear regression of AU/mL by age, BMI and gender.

	Beta (95% CI)	*p* Value
AGE (≤47 vs. >47 years)	0.292 (0.111; 0.473)	0.002
BMI (underweight/normal vs. pre-obesity/obesity)	0.307 (0.114; 0.501)	0.002
GENDER (female vs. male)	0.075 (−0.112; +0.262)	0.43

## Data Availability

Data is going to be added to the clinical trial registry (ISRCTN55371988) where they will be available.
